# A randomized clinical trial of technical modifications of appendix stump closure during laparoscopic appendectomy for uncomplicated acute appendicitis

**DOI:** 10.1186/s12893-021-01279-z

**Published:** 2021-05-31

**Authors:** Peter Ihnát, Milan Tesař, Lubomír Tulinský, Lucia Ihnát Rudinská, Okaikor Okantey, Štefan Durdík

**Affiliations:** 1 Department of Surgery, University Hospital Ostrava, 17.listopadu 1790, Ostrava, 708 52 Czech Republic; 2 Department of Forensic Medicine, University Hospital Ostrava, 17.listopadu 1790, Ostrava, 708 52 Czech Republic; 3 Department of Cardiovascular Surgery, University Hospital Ostrava, 17.listopadu 1790, Ostrava, 708 52 Czech Republic; 4 Department of Oncosurgery, Faculty of Medicine, Commenius University Bratislava, Spitalska 24, Bratislava, 813 72 Slovakia

**Keywords:** Appendicitis, Laparoscopic appendectomy, Appendix stump closure, Postoperative complications, Cost-effectiveness

## Abstract

**Background:**

Closure of the appendix stump presents the most critical part of laparoscopic appendectomy. The aim of the present study was to compare the medical outcomes and cost analysis of laparoscopic appendectomy with respect to the different methods of stump closure.

**Methods:**

This was a prospective randomized clinical trial conducted in a single institution (University Hospital Ostrava) within a 2-year study period. All included patients were randomized into one of three trial arms (endoloop, hem-o-lok clips or endostapler).

**Results:**

In total, 180 patients (60 patients in each arm) were enrolled into the study. The mean length of hospital stay (3.6 ± 1.7 days) was comparable in all study arms. The shortest operative time was noted in the *hem-o-lok* subgroup of patients (37.9 ± 12.5 min). Superficial surgical site infection was detected in 4.4% of study patients; deep surgical site infection was noted in 1.7% of the patients. The frequency of surgical site infections was comparable in all study arms (p = 0.7173). The mean direct costs of laparoscopic appendectomy were significantly the lowest in the *hem-o-lok* subgroup of patients. Laparoscopic appendectomy is not a profit-making procedure in our institution (mean profit of made from the study patients was—104.3 ± 579.2 Euro). Closure of the appendix stump by means of endostapler presents the most expensive and the highest loss-incurring technique (p = 0.0072).

**Conclusions:**

The present study indicates that all technical modifications of appendix stump closure are comparable with regards to postoperative complications. The stapler technique is significantly the most expensive. We concluded that hem-o-lok clips have the potential for becoming the preferred method of securing the appendix base during laparoscopic appendectomy. *Trial registration* NCT03750032 **(**http://www.clinicaltrials.gov).

## Introduction

Acute appendicitis is the most common inflammatory disease of the abdominal cavity requiring acute abdominal surgery [1, 2]. Laparoscopic appendectomy (L-APPE) has become the gold standard with respect to the treatment of acute appendicitis in many institutions nowadays [3–5]. L-APPE has several advantages over open appendectomy such as reduced postoperative pain, faster recovery, better cosmetic results and reduced wound infection rate [4, 6, 7].

Closure of the appendix stump presents the most critical part of L-APPE; effective and safe closure is important to prevent serious postoperative complications such as abscesses, peritonitis and sepsis [1, 8, 9]. Moreover, the technique of appendix stump closure presents one of the principal factors determining the cost of the procedure [8, 10]. Several technical modifications of stump closure during L-APPE are currently available—closure with a clip, closure using an endoloop or stapler [8, 9, 11, 12].

There are ongoing controversies regarding the technique of appendix stump closure; however, an optimal method remains unclear. Furthermore, our literature search revealed that available data pertaining to evidence-based medicine regarding this topic are highly insufficient [1, 9]. According to the recent Cochrane systematic review, there are only 8 randomized trials available (encompassing 850 patients) focused on the assessment of techniques employed in appendix stump closure during L-APPE [9]. We conducted a prospective randomized single-center trial primarily focused on the comparison of the different techniques of appendix stump closure. The aim of the present clinical trial was to compare medical outcomes and costs of laparoscopic appendectomy with different methods of appendix stump closure (endostapler, endoloop and Hem-o-lok clips).

## Material and methods

### Design and setting

This was a prospective randomized clinical trial designed to evaluate the medical outcomes and cost analysis of patients undergoing laparoscopic appendectomy with different methods of appendix stump closure. The trial was conducted in the University Hospital Ostrava, Czech Republic. All patients with clinical signs of acute appendicitis undergoing L-APPE at University Hospital Ostrava within a study period (1st January 2018–31st December 2019) were assessed for study eligibility. The study was approved by ethics committee of the University Hospital Ostrava (ref. number 449/2018) and performed in accordance with the ethical standards of the Declaration of Helsinki (1964) and its subsequent amendments. Written informed consent were obtained from all included patients; anonymity was ensured. The trial was registered on http://www.clinicaltrials.gov (trial identifier NCT03750032).

The inclusion criteria were age ≥ 18 years, clinical signs of acute appendicitis and suitability for laparoscopic surgery. The exclusion criteria were peroperative findings of necrosis or advanced inflammatory changes in the area of the appendix stump and conversion to laparotomy due to diffuse peritonitis. Incomplete data in patients lost to follow-up were the reason to exclude these patients from the study analysis. All participating surgeons were instructed to exclude from the study (irrespective of trial allocation) patients who exhibited peroperative findings of necrosis or advanced inflammatory changes, which would prevent a safe appendectomy with endoloop or clips.

Within the study period, each study subject was randomized to one of 3 trial arms (endoloop, Hem-o-lok or stapler) using an envelope method (all envelopes were prepared in advance, with a ratio of 1:1:1). Randomization was performed before the surgery; patients with peroperative findings of necrosis or advanced inflammatory changes (as stated in the exclusion criteria) were excluded from the study, the envelope was sealed again and returned to the basket. Similarly, the envelopes of patients lost to follow-up were returned to the randomisation pool. “Blinding” was not involved in our study design, because postoperative quality of life or aesthetic outcomes of the surgery were not assessed.

There were two limitations of our study regarding sample size: (1) the number of laparoscopic appendectomies performed in our institution each year, and (2) the limited study period of two years due to funding regulations of our study. As a result of these limitations, the trial sample size of 180 patients (60 patients in each trial arm) was determined. The operative time, intraoperative and postoperative complications were the primary outcome measures of the study, economical outcomes were the secondary outcome measures.

### Surgical technique

In all the study patients, L-APPE was performed by certified surgeons experienced in advanced laparoscopic surgery. Surgery was performed under general anaesthesia; the patient was placed in the supine, Trendelenburg position. The 3-port technique (a 10-mm camera port in periumbilical region, a 5-mm port in the left lower quadrant and a 10-mm port in suprapubic region) was employed. After proper abdominal cavity exploration, the decision to perform appendectomy was made. The mesoappendix was dissected using a harmonic scalpel.

In the first group, the appendix base was secured using a total of three vicryl endoloops (two loops on the appendix base and one on the distal part which would be removed). In the second group, three Hem-o-lok clips size XL (Hem-o-lock, Weck Closure Systems, Research Triangle Park, NC, USA) were used to secure the appendix base (two clips on the appendix base and on the distal part of the appendix). In the third group, the 10-mm port in a suprapubic region was changed for a 12-mm port and the appendix base was dissected by means of a 45 mm stapling device (Ethicon, Endosurgery, Cincinnati, OH).

In all patients, the appendix was inserted into the protective plastic bag and removed via the suprapubic port. The appendix stumps were not invaginated in our study patients; drainage of the abdominal cavity was performed according to the preference of the operating surgeon. The decision to drain the abdominal cavity was done after extraction of the appendix, mostly in patients with gangrenous appendicitis. Broad-spectrum antibiotics were administered in patients with gangrenous appendicitis for 7 days.

### Data collection

All data were collected prospectively during the study. The demographic and clinical data of all study patients (age, gender, BMI, ASA classification) were recorded into a study database. The intraoperative complications and operative time were assessed on the 1st day after the surgery. The postoperative complications were evaluated during patient follow-up at the outpatient surgical department one month after the surgery. The postoperative surgical complications were graded according to the Clavien-Dindo Classification [13]. The follow-up of the study patients ended a month after surgery. Data regarding cost analysis of L-APPE of all study patients were extracted from the hospital economic database and recorded into a study database. The direct costs of L-APPE were calculated as the sum of all expenses associated with every diagnostic and therapeutic procedure which had been realized during patient’s hospital stay (costs of laboratory and imaging examinations, operating room time, costs of anaesthesia and surgery including all used medical devices, cost of hospital stay). The costs of surgical devices (stapler, endoloop, clips, 12-mm port, endobag) were calculated as the true cost paid by the hospital (the real prices which the hospital paid to the provider). The costs of hospital stay were calculated using an average cost of hospitalization per day. For all study patients, the hospital received payments from health insurance companies based on the current healthcare payment system CZ-DRG (Czech Diagnosis—Related Group system).

### Statistical analysis

The acquired data underwent analysis by means of descriptive statistics. The differences between the subgroups were tested using a Chi-square test for categorical variables, Anova (Analysis of Variance) and Kruskal–Wallis rank sum test for continuous variables (relative frequencies). The statistical analysis was conducted using STATISTICA 10. A level of significance of α = 0.05 was considered statistically significant.

## Results

**Fig. 1:**
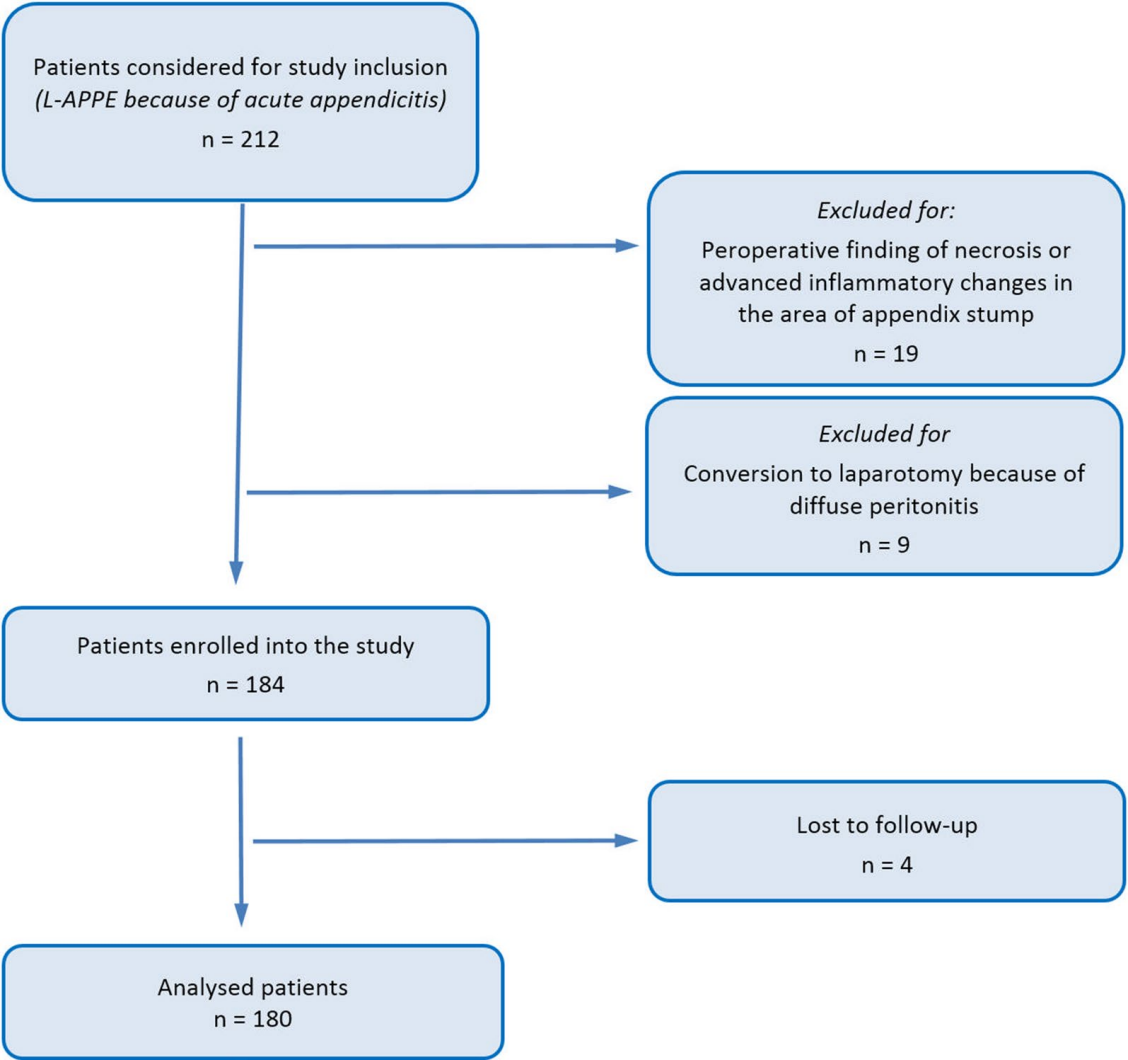
Study flow-chart diagram

In total, 212 patients underwent L-APPE at University Hospital Ostrava within the study period. Of these, 28 patients (13.2%) were excluded due to the study design and exclusion criteria and 4 patients (1.9%) were lost to follow-up (Fig. 1). There were 19 patients excluded due to peroperative findings of necrosis or advanced inflammatory changes in the area of appendix stump—surgery was completed laparoscopically in all these patients (laparoscopic appendectomy using a stapler was performed in 84.2% of patients, laparoscopic ileocecal resection in 15.8% of these patients). 9 patients were excluded due to the conversion to laparotomy upon the discovery of severe diffuse peritonitis peroperatively. In total, 180 study patients underwent analysis.

The mean age of study patients was 35.8 ± 16.9 years (mean ± SD); there were 103 (57.2%) women and 77 (42.8%) men. The vast majority of patients were preoperatively classified as ASA class I or II; the mean BMI was 25.7 ± 4.2 kg/m^2^. Detailed data regarding demographics and clinical characteristics of the study patients are presented in Table 1. The most frequent type of inflammation was phlegmonous appendicitis (62.2% of study patients).

**Table 1:** Demographics and clinical data of study patients

Parameter	Endoloop (n = 60)	Hem-o-lok (n = 60)	Stapler (n = 60)	P-value	Total (n = 180)
Age (years, mean ± SD)	31.1 ± 11.7	38.9 ± 16.3	46.1 ± 18.6	< 0.001	35.8 ± 16.9
Gender, n (%)					
Female	28 (46.7)	36 (60.0)	39 (65.0)	0.1106	103 (57.2)
Male	32 (53.3)	24 (40.0)	21 (35.0)		77 (42.8)
BMI (kg/m^2^, mean ± SD)	24.5 ± 3.9	26.4 ± 4.3	26.1 ± 4.3	0.0319	25.7 ± 4.2
ASA classification, n (%)					
I	38 (63.3)	26 (43.3)	25 (41.7)	0.1072	89 (49.4)
II	20 (33.3)	29 (48.3)	29 (48.3)		78 (43.3)
III	2 (3.3)	5 (8.3)	6 (10.0)		13 (7.2)
Appendicitis type, n (%)					
Catarrhal	21 (35.0)	20 (33.3)	5 (8.3)	0.0026	46 (25.6)
Phlegmonous	31 (51.7)	32 (53.3)	49 (81.7)		112 (62.2)
Gangrenous	8 (13.3)	8 (13.3)	6 (10.0)		22 (12.2)

*SD* standard deviation, *BMI* body mass index, *ASA* American Society of Anaesthesiologist

Data regarding perioperative outcomes (operative time, hospital stay, intraoperative and postoperative complications) are presented in Table 2. The mean operative time was 42.0 ± 13.0 min. The shortest time was noted in the *hem-o-lok* subgroup of patients (37.9 ± 12.5 min); the differences in operative times between study subgroups were statistically significant (p = 0.0088). The mean length of hospital stay (3.6 ± 1.7 days) was comparable in all our study subgroups (p = 0.969).

**Table 2:** Operative time, hospital stay and postoperative complications of study patients

Parameter	Endoloop (n = 60)	Hem-o-lok (n = 60)	Stapler (n = 60)	*P*-value	Total (n = 180)
Operative time (minutes, mean ± SD)	45 ± 12.0	37.9 ± 12.5	42.9 ± 13.4	0.0088	42.0 ± 13.0
Hospital stay (days, mean ± SD)	3.6 ± 1.8	3.6 ± 1.7	3.7 ± 1.5	0.969	3.6 ± 1.7
Intraoperative complications, n (%)	0 (0.0)	0 (0.0)	7 (11.7)		7 (3.9)
Postoperative complications, n (%)					
Superficial SSI	4 (6.7)	3 (5.0)	1 (1.7)	0.7173	8 (4.4)
Deep SSI	1 (1.7)	1 (1.7)	1 (1.7)		3 (1.7)

*SD* standard deviation, *Superficial SSI* superficial surgical site infection, *Deep SSI* deep surgical site infection

There were 7 (11.7%) intraoperative complications in the *stapler* subgroup of patients. All these complications (bleeding from the stapler line) were easy to manage surgically—via application of metal clip. No intraoperative complications were noted in the *endoloop* and *hem-o-lok* subgroups.

A 30-day postoperative morbidity was manifested in 6.1% of the study patients; all postoperative complications were surgical. The prevalence of postoperative complications in the study subgroups was comparable (p = 0.7173). In 8 (4.4%) patients, superficial SSI (Surgical Site Infection) was detected and classified as grade I according to Clavien-Dindo classification. All superficial SSIs were of wounds localised in the suprapubic region. Deep SSI was noted in 3 (1.7%) patients and classified as grade IIIb Clavien-Dindo classification. There was no mortality reported in our study group. There were no reoperations or readmissions to hospital during a 30-day postoperative period in our study patients.

Data regarding costs of L-APPE are presented in Table 3. The mean direct costs of L-APPE (costs of the diagnostic process, surgery and postoperative hospital care) were 1816.1 ± 624.3 Euro. The highest costs were recorded in the *stapler* subgroup of patients while the lowest costs, in the *hem-o-lok* subgroup. The differences in direct costs between the study subgroups were statistically significant (p < *0.001*). As clearly stated in Table 3, L-APPE is not a profit-making procedure in our institution (the mean profit of study patients was—104.3 ± 579.2 Euro). The closure of the appendix stump by means of endostapler presents the most expensive and highest loss-incurring technique (p = 0.0072).

**Table 3:** Cost-effectiveness of L-APPE in our study patients

Parameter	Endoloop (n = 60)	Hem-o-lok (n = 60)	Stapler (n = 60)	*P*-value	Total (n = 180)
Direct costs (Euro, mean ± SD)	1705.4 ± 500.9	1624.9 ± 768	2120 ± 435	< 0.001	1816.1 ± 624.3
Payments from health insurance companies (Euro, mean ± SD)	1603.7 ± 227	1619.8 ± 355.6	1910.6 ± 350	< 0.001	1711.8 ± 346
Profit (Euro, mean ± SD)	− 98.6 ± 487.4	− 5.1 ± 761	− 209.3 ± 412	0.0072	− 104.3 ± 579.2

## Discussion

L-APPE presents the preferred surgical technique for the treatment of acute appendicitis in many institutions. The advantages of laparoscopic approach are indisputable—better exploration of the abdominal/pelvic cavity, reduced surgical trauma, faster recovery rates, reduced rate of SSI and better cosmetic results [1, 4, 6, 7, 14]. The availability of L-APPE depends on the economic status of the country. It has been demonstrated that the laparoscopic approach is performed more frequently in high-income countries in comparison with low-income countries (67.7% vs. 8.1%) and it is associated with better postoperative outcomes (significantly less SSI rates in high-income countries) [15].

All L-APPE were performed by certified laparoscopic surgeons in all our study patients. Operating surgeons were experienced in all three techniques of appendix stump closure. For this reason, we are convinced that the level of experience of the participating surgeons had no influence on our study outcomes.

There were statistically significant differences in clinical parameters between our study subgroups (age, BMI and type of appendicitis). The patients in the *stapler* subgroup were older in comparison with patients in the other 2 subgroups, patients in the *hem-o-lok* subgroup had the highest BMI and phlegmonous appendicitis was detected in the largest proportion of patients in the *stapler* subgroup. The allocation of patients into the study subgroups was as a result of the randomization process. However, all participating surgeons were instructed to exclude from the study (irrespective of trial allocation) patients with peroperative findings of necrosis or advanced inflammatory changes of appendix base. This exclusion criterion might cause a relevant selection bias. It can also be supposed that a larger study sample size would nullify the differences in the clinical parameters between the study subgroups. The main outcome of our study was that all technical modifications of appendix stump closure are comparable with regards to the postoperative complications. Regardless of higher BMI or higher proportion of patients with phlegmonous appendicitis, the postoperative morbidity in all study subgroups was similar.

The number of postoperative surgical complications after L-APPE was very low in all our study subgroups. Besides superficial SSI in 4.4% of patients, we noted serious complications requiring surgical intervention in only 1.7% of the study patients (grade IIIb Clavien-Dindo classification). In the available literature, the rates of surgical re-interventions after L-APPE vary from 1.2 to 6.0% of patients [5, 9, 12, 15–19]. We did not detect any non-surgical postoperative complications in our study group. We suppose that this is resultant of two actualities—demographic/clinical characteristics of our study group (many young patients with minimal co-morbidities) and very minimal surgical trauma during L-APPE associated with fast recovery rates.

Closure of the appendix stump presents the most critical and controversial part of L-APPE. The technique of appendix stump closure has an impact on postoperative complications and significantly affects the cost of the procedure [1, 9, 12]. Several researchers have conducted clinical studies to examine the different methods of appendix stump closure, but an optimal technique is yet to be determined. The Endoloop was one of the first methods used in stump closure. The main problem of endoloop technique is the risk of leaving the knot loose (the surgeon may be afraid to pull the endoloop more due to the thread cutting through swollen appendix base). Appendix stump closure with clips (metal, plastic, absorbable polymeric) may be limited by the diameter of appendix base and there is a risk of clip opening and sliding off. Hem-o-lok clips have the advantage of securing the appendix stump with a minimal risk of sliding off. Using the endostapler is more comfortable but the cost of the stapler is much higher in comparison with the other methods. For this reason, hem-o-lok clips seem to be a reasonable solution that is both technically easy and cost-effective [12, 16, 20].

Our trial demonstrated similar postoperative morbidity after L-APPE with different methods of appendix stump closure. The most important postoperative complication directly associated with the technique of stump closure (deep SSI) was noted in 1.7% of patients in all study subgroups. Therefore, we conclude that there are no differences in terms of the safety of stump closure between endoloop, hem-o-lok and stapler. However, it must be emphasized that our study sample size was limited (under-powered study can be the cause of selection bias, which can lead to compromised study outcomes).

One of the most important prospective studies focused on the different techniques of appendix stump closure was published recently by Delibegović et al. The design of the study was similar to ours (30 patients endoloop, 30 patients hem-o-lok and 30 patients stapler and 30 patients DS clip). The authors claimed zero postoperative morbidity in study patients and confirmed equality of the different techniques [12]. Matyja et al. compared appendix stump closure by means of a clip, stapler or laparoscopic suture (20 patients in each study arm) and found no differences in postoperative morbidity [8].

Muñoz-Cruzado et al. conducted a retrospective cohort study of 709 patients undergoing L-APPE with different techniques of appendix stump closure [19]. Authors found no differences in postoperative complications between patients with uncomplicated appendicitis. In patients with complicated appendicitis, higher postoperative morbidity and higher number of reoperations were noted in endoloop subgroup compared to stapler subgroup of patients.

Data from Polish multicenter cohort study (1269 patients from 18 surgical unites) revealed superior outcomes of endostapler in terms of overall postoperative morbidity and length of hospital stay. However, authors conclude that clinical benefits of staplers for appendix stump closure are based on a non-randomized group of patients and are therefore prone to selection bias [18].

The shortest operative time was recorded in the *hem-o-lok* subgroup of our study patients (37.9 ± 12.5 min). The operative time differences could have been influenced by uneven distribution of severe inflammations between our study subgroups. According to the data in available literature, the endoloop and laparoscopic suture present the most time-consuming methods of appendix stump closure. Furthermore, laparoscopic suture is the most technically demanding method which requires experience in laparoscopic suturing [8, 17, 20]. Some authors referred shorter operative times of L-APPE when using the stapler for appendix transection, others published shorter operative times using clips [8, 12, 21–25].

Different techniques of appendix stump closure have been carefully analyzed also in the 2020 update of the WSES (World Society of Emergency Surgery) Jerusalem Guidelines [26]. Authors of the Jerusalem consensus on diagnosis and treatment of acute appendicitis conclude that there are no clinical advantages in the use of endostaplers over endoloops for stump closure for both adults and children in either simple or complicated appendicitis, except for a lower incidence of wound infection when using endostaplers in children with uncomplicated appendicitis. Polymeric clips may be the cheapest and easiest method (with shorter operative times) for stump closure in uncomplicated appendicitis [26].

The secondary aim of the present study was to review the costs of L-APPE in our institution (tertiary teaching hospital) and to specify the loss/profit made under the current economic conditions in the Czech Republic with respect to the medical devices used. The analysis (direct costs of provided healthcare of all study patients versus payments received from health insurance companies) revealed that L-APPE is unequivocally a loss-incurring procedure in our institution regardless of the technique used for appendix stump closure. As expected, appendix transection by the stapler presents the most expensive method; the differences in costs between our study subgroups were statistically significant. The difference in the costs of surgical devices (endoloop vs. hem-o-lok clips vs. stapler) presented a principal factor determining the difference in direct costs between the study groups. However, there were also some minor factors affecting the difference in direct costs such as differences in operating theatre time and length of hospital stay.

The present study was focused on the investigation of medical outcomes and cost analysis of L-APPE with different methods of appendix stump closure. The strengths of the study are: study design (prospective controlled randomized clinical trial), the standardized surgical technique performed by experienced laparoscopic surgeons in a tertiary teaching hospital and the precise assessment of medical outcomes and cost analysis. Nevertheless, the study had several limitations: the study sample size was not calculated by a statistician prior to the study, the limited sample size could have been the cause of selection bias and the data regarding costs reflects the current economic situation in healthcare system of the Czech Republic (external validity of this data is very limited). However, to the best of our knowledge, this is the largest trial investigating the technical modifications of appendix stump closure during L-APPE.

*In conclusion, L-APPE presents* a well-established surgical technique in the treatment of acute appendicitis. According to our study outcomes, all technical modifications of appendix stump closure are comparable with regards to postoperative complications. The operative time is significantly longer when the endoloop is employed for stump closure; the stapler technique is significantly the most expensive. Taking all these facts into account, hem-o-lok clips seem to have the potential for becoming the preferred method of securing the appendix base during L-APPE.

## Data Availability

The datasets used and/or analysed during the current study are available from the corresponding author on reasonable request.
